# Sociodemographic and sex differences in the global burden of hypertensive heart disease, 1990–2021: a population-based analysis

**DOI:** 10.1186/s41182-025-00791-9

**Published:** 2025-08-14

**Authors:** Fangfei Nie, Xiaorong Wang, Airong Yang, Jiaolong He, Jie Bai, Ping Yan, Xiaozhou Wang

**Affiliations:** 1 Clinical Medical College, Qinghai University, Xining, Qinghai China; 2 Qinghai Cardio-Cerebrovascular Specialty Hospital, Xining, Qinghai China; 3 Department of Critical Care Medicine, First Affiliated Hospital of Jishou University, Jishou, Hunan China

**Keywords:** Hypertensive heart disease, Global burden, Socio-demographic index, Age-standardized DALY rate, EAPC, Sex differences

## Abstract

**Background:**

Hypertensive heart disease (HHD) is a major global contributor to cardiovascular-related disability. Although its burden has been previously described, detailed analyses of long-term disability trends by sociodemographic level and sex remain scarce. This study aimed to systematically assess global and subgroup-specific patterns in HHD-related disability-adjusted life years (DALYs) from 1990 to 2021.

**Methods:**

Data from the Global Burden of Disease Study 2021 were used to evaluate age-standardized DALY rates for HHD across five socio-demographic index (SDI) levels and by sex from 1990 to 2021. Temporal trends were assessed using Joinpoint regression with estimated annual percent change (EAPC). Future rates through 2036 were projected using Bayesian age–period–cohort models. Decomposition analysis quantified contributions of population growth, aging, and epidemiologic changes, and risk-attributable DALYs were estimated for five modifiable exposures.

**Results:**

Globally, DALYs from HHD increased from 15.47 to 25.46 million (+ 64.6%) between 1990 and 2021, while the age-standardized DALY rate declined from 406.51 to 301.58 per 100,000 population (EAPC – 0.96; 95% CI – 0.98 to – 0.93). DALY counts rose and age-standardized DALY rate declined across all SDI levels, with the greatest reduction in middle-SDI regions (EAPC – 1.89; 95% CI – 2.17 to – 1.60) and notable decreases in high-middle SDI regions (EAPC – 1.06; 95% CI –1.68 to – 0.43). Rates in females remained consistently higher than in males, with projections suggesting persistent disparities through 2036. Decomposition analysis indicated that population aging and growth were the main contributors to DALY increases, partially offset by epidemiological improvements. High systolic blood pressure (− 100%) and elevated body mass index (− 50%) were the leading modifiable risk factors across SDI levels.

**Conclusions:**

Despite declines in age-standardized DALY rate, the absolute HHD burden continues to grow, particularly among women and in low-SDI regions. Targeted, equity-focused cardiovascular strategies are urgently needed to address these persistent disparities.

**Supplementary Information:**

The online version contains supplementary material available at 10.1186/s41182-025-00791-9.

## Introduction

Hypertensive heart disease (HHD) commonly arises from prolonged hypertension and is characterized by cardiac structural abnormalities such as left ventricular hypertrophy and diastolic dysfunction  [1]. These changes may gradually progress, contributing to myocardial remodeling and, in advanced stages, heart failure  [2]. Over the past three decades, the global burden of HHD has increased markedly, largely due to demographic aging and suboptimal hypertension management  [3]. Environmental factors also play a role; for instance, household air pollution has been linked to elevated blood pressure  [4], and lead exposure is an established cardiovascular risk factor  [5].

Although previous studies using Global Burden of Disease (GBD) 2019 data have explored the prevalence and disability-adjusted life years (DALYs) associated with HHD  [6], stratified analyses by sex and socio-demographic index (SDI) remain limited. Most prior investigations have focused on prevalence or mortality  [7], and only recently have researchers begun to apply age-standardized DALY rates to better capture the burden of chronic cardiovascular conditions  [8]. Subgroup-specific analyses are scarce  [9], and advanced methods such as Bayesian age–period–cohort (BAPC) models and decomposition techniques have seldom been applied to HHD  [10]. These advanced methods allow for more precise assessment of temporal dynamics, identification of age and cohort effects, and dissection of demographic contributions to the overall disease burden. Moreover, the contribution of modifiable risk factors beyond systolic blood pressure—such as body mass index (BMI), sodium intake, alcohol use, and lead exposure—has not been fully examined across SDI strata and between sexes  [11]. As a result, current evidence often reflects national-level aggregates and lacks attention to underlying disparities in disease burden. Recent GBD-based studies have primarily focused on national or regional mortality trends and have not comprehensively assessed global DALY patterns stratified by SDI and sex. Controversies remain regarding the contributions of demographic versus epidemiological drivers in shaping HHD burden, and whether improvements in blood pressure control are effectively reducing disease burden worldwide. Furthermore, evidence gaps persist in understanding sex-specific differences and the role of emerging metabolic and environmental risk factors across diverse socioeconomic contexts. Addressing these gaps is crucial to inform targeted interventions and reduce global health disparities.

To address these gaps, we analyzed data from the GBD 2021 study to assess global and SDI-stratified trends in the DALY burden of HHD from 1990 to 2021. We applied BAPC modeling to forecast future trajectories through 2036 and performed decomposition analysis to disentangle demographic and epidemiological contributions. We further estimated DALYs attributable to five modifiable risk factors in 2021. These complementary approaches were used to generate evidence that may support more targeted, equity-oriented cardiovascular prevention strategies, particularly in high-burden settings.

## Methods

### Study design and data source

We conducted a secondary analysis based on the GBD 2021, which estimates 371 diseases and 88 risk factors across 204 countries and territories from 1990 to 2021 [12]. This study followed GATHER guidelines and used publicly available, de-identified summary datasets stratified by age, sex, year, and location [13]. All data were obtained through the Global Health Data Exchange (GHDx) portal (http://ghdx.healthdata.org/gbd-2021). Although GBD also groups countries into seven super regions for certain analyses, our study focused on global and SDI-based estimates. The definitions and member countries of these super regions are provided in Supplementary Table S1. GBD estimates used in this study were produced by the Institute for Health Metrics and Evaluation (IHME) using standardized, peer-reviewed modeling approaches (e.g., CODEm and DisMod-MR 2.1). Detailed descriptions of these models and their assumptions are available in the GBD 2021 technical appendix and related publications. Our analysis used publicly available summary estimates without independent recalibration or re-modeling.

### Outcome definitions and metrics

Hypertensive heart disease (HHD) was defined using the International Classification of Diseases, 10th Revision (ICD-10) codes I11–I11.9, in alignment with the Global Burden of Disease (GBD) 2021 cause list [14]. The primary outcome measured was disability-adjusted life years (DALYs), calculated as the sum of years of life lost (YLLs) and years lived with disability (YLDs) [15]. We assessed both the absolute counts of DALYs and the age-standardized DALY rate per 100,000 population. Age-standardized DALY rates were calculated using the GBD world standard population, which is a composite age structure developed to facilitate consistent comparisons across countries and over time. This standardization removes the effects of differences in population age distributions, allowing for more accurate assessment of trends and comparisons between regions.

### Trend and Joinpoint analysis

Temporal trends from 1990 to 2021 were evaluated using the estimated annual percentage change (EAPC), derived from log-linear regression models based on age-standardized DALY rates [16]. Joinpoint regression was used to detect inflection points and annual percentage change (APC) within defined segments [17]. Analyses were stratified by sex and socio-demographic index (SDI). Confidence intervals (CIs) for EAPCs and DALY rates were calculated using log-linear regression models, with standard errors of the regression coefficients reflecting statistical uncertainty. These CIs indicate the precision of the estimates and help assess whether observed trends are statistically significant.

### Socio-demographic index (SDI)

We assessed the cross-sectional relationship between SDI and HHD burden in 2021 using a scatter plot of DALY rates *versus* SDI for 204 countries and territories. Country names followed GBD standardization. A LOESS regression curve was applied to visualize non-linear associations, implemented via the R package ggplot2 [function: geom_smooth (method = “loess”)] [18].

### Projection modeling

Projections for DALY rates from 2022 to 2036 were conducted using a BAPC model with second-order random walk (RW2) priors to account for autocorrelation across time dimensions [19]. Posterior median estimates and 95% credible intervals were obtained. This approach has been previously applied in GBD-based forecasting analyses [20].

### Decomposition and risk attribution

We used the Das Gupta method to decompose changes in total DALYs between 1990 and 2021 into components attributable to population growth, population aging, and changes in age-specific DALY rates [21]. Risk-attributable DALYs in 2021 were estimated using the GBD comparative risk assessment framework, including five modifiable factors: high systolic blood pressure, high body mass index, high sodium intake, high alcohol use, and lead exposure. Attributable fractions and DALYs were stratified by sex and SDI level [22].

### Statistical tools

All analyses were conducted using R (version 4.4.3). Data visualization employed base R and ggplot2. Bayesian modeling was implemented with the BAPC and INLA packages. For GBD-derived estimates, 95% uncertainty intervals (UIs) were generated from 1,000 posterior draws, with the 2.5th and 97.5th percentiles representing the lower and upper bounds. UIs capture both sampling variability and model uncertainty. Together, CIs and UIs provide complementary perspectives on the precision and uncertainty of our results and should be interpreted accordingly. As the GBD framework already incorporates simulation-based uncertainty intervals, no additional hypothesis testing was performed.

## Results

### Global and SDI-based DALY trends in HHD burden

Between 1990 and 2021, the global number of DALYs due to HHD increased from 15.47 million to 25.46 million, whereas the age-standardized DALY rate declined from 406.51 to 301.58 per 100,000 population (EAPC – 0.96; 95% CI – 0.98 to – 0.93) (Table 1). By SDI level, high-SDI regions experienced an increase in DALYs from 1.70 to 3.09 million, while the age-standardized DALY rate remained relatively stable. High-middle SDI regions saw a rise in DALYs from 2.89 to 4.39 million, accompanied by a marked decline in the age-standardized DALY rate from 315.23 to 228.02 (EAPC – 1.06; 95% CI – 1.68 to – 0.43). Middle-SDI regions showed an increase in DALYs from 6.00 to 8.93 million, alongside a substantial age-standardized DALY rate reduction from 645.06 to 355.67 (EAPC – 1.89; 95% CI – 2.17 to – 1.60). In low-middle SDI regions, DALYs rose from 3.11 to 5.96 million, with the age-standardized DALY rate declining from 545.44 to 412.17. Low-SDI regions reported an increase in DALYs from 1.75 to 3.06 million, along with a decrease in the age-standardized DALY rate from 817.25 to 640.71.

**Table 1:** Global and SDI burden of hypertensive heart disease (HHD) from 1990 to 2021: disability-adjusted life years (DALY) and temporal trends

Location	DALYs case (95% UI)		DALYs rates/100,000 (95% UI)		EAPC of DALYs rates (95% CI)	Age-standardized DALYs rate/100,000 (95% UI)		EAPC of age-standardized DALYs rates (95% CI)
	1990	2021	1990	2021	1990–2021	1990	2021	1990–2021
Global	15473830 (12310725–17311822)	25462185 (21493312–28047521)	290.12 (230.81–324.58)	322.66 (272.37–355.42)	0.35 (− 0.15 to 0.84)	406.51 (328.94–452.24)	301.58 (255.06–332.06)	− 0.96 (− 0.98 to − 0.93)
SDI								
High SDI	1695962 (1583912–1767114)	3098667 (2717718–3397624)	192.83 (180.09–200.91)	283.23 (248.41–310.56)	1.26 (0.91 to 1.62)	155.64 (145.29–162.29)	149.35 (134.5–162.92)	− 0.16 (− 0.87 to 0.56)
High-middle SDI	2891667 (2552953–3233035)	4389745 (3890115–4989434)	271.89 (240.04–303.99)	336.63 (298.31–382.62)	0.69 (0.46 to 0.92)	315.23 (278.30–350.96)	228.02 (201.54–258.50)	− 1.06 (− 1.68 to − 0.43)
Middle SDI	6002937 (4223843–6811307)	8930493 (6909201–10413694)	348.42 (245.16–395.34)	364.73 (282.18–425.30)	0.15 (− 0.05 to 0.35)	645.06 (459.80–729.11)	355.67 (273.61–414.56)	− 1.89 (− 2.17 to − 1.60)
Low-middle SDI	3111599 (2200759–3753608)	5955402 (4908448–6812461)	267.92 (189.49–323.19)	310.00 (255.50–354.61)	0.47 (− 0.43 to 1.37)	545.44 (390.96–652.79)	438.48 (364.14–503.27)	− 0.70 (− 1.55 to 0.15)
Low SDI	1753192 (1128631–2257732)	3055862 (2148436–3826137)	349.73 (225.14–450.37)	273.48 (192.27–342.42)	− 0.84 (− 2.36 to 0.71)	817.25 (545.80–1035.65)	640.71 (451.46–786.79)	− 0.84 (− 2.46 to 0.80)

SDI: socio-demographic index; EAPC: estimated annual percentage change; UI: uncertainty interval; CI: confidence interval

### Temporal trends in age-standardized DALY rates from 1990 to 2021

**Fig. 1:**
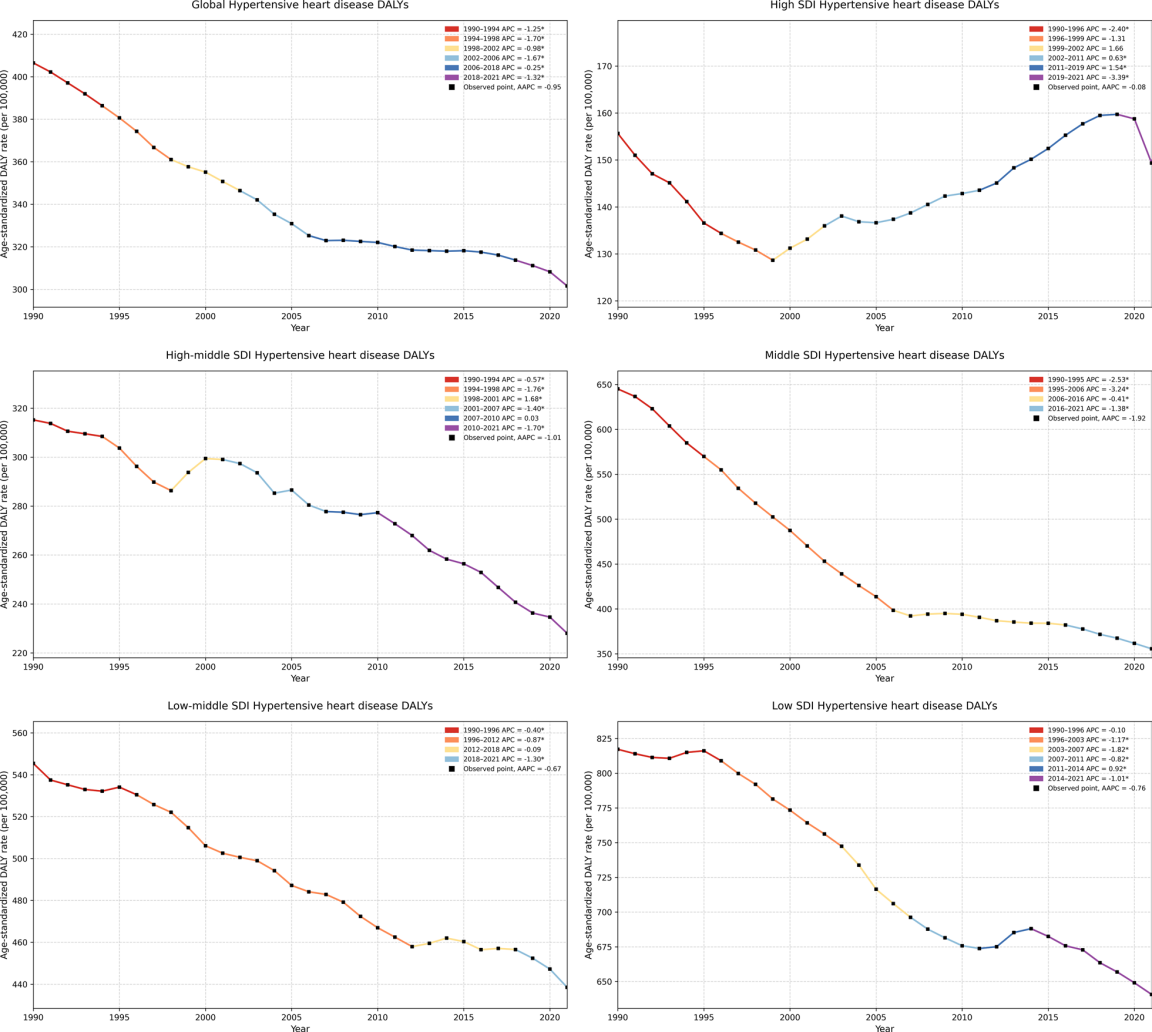
Trends in disability-adjusted life years (DALYs) due to hypertensive heart disease (HHD) from 1990 to 2021 by global and SDI level. Joinpoint regression analysis of temporal trends in age-standardized DALY rates for HHD (1990–2021), stratified globally and by SDI level. Segments indicate annual percentage change (APC); asterisks denote *p* < 0.05. AAPC summarizes overall trends within each group

From 1990 to 2021, the global age-standardized DALY rate for HHD exhibited a general downward trend with distinct segmented variations, as revealed by Joinpoint regression identifying five inflection points (Fig. 1). A sharp decline was observed from 1990 to 1994 (APC – 1.25), followed by continued decreases from 1994 to 1998 (APC – 1.70) and 1998 to 2002 (APC – 0.98). The rate declined further between 2002 and 2006 (APC – 1.67), plateaued from 2006 to 2018 (APC – 0.25), and experienced a modest decrease again from 2018 to 2021 (APC – 1.32). Trends varied across SDI levels. Middle- and high-middle SDI regions exhibited the most sustained reductions in age-standardized DALY rates, with middle SDI countries showing the steepest overall decline. In high-SDI settings, age-standardized DALY rate dropped sharply between 1990 and 1996 (APC – 2.40), slowed after 2006, and temporarily rose between 2012 and 2018 (APC  + 1.94), before declining again after 2018 (APC – 0.89). In contrast, low-middle and low-SDI regions experienced persistently high age-standardized DALY rate, with gradual and sometimes reversed trends—most notably, a short-term increase in low-SDI regions between 2013 and 2017.

### Cross-sectional association between age-standardized DALY rate and SDI in 2021

**Fig. 2:**
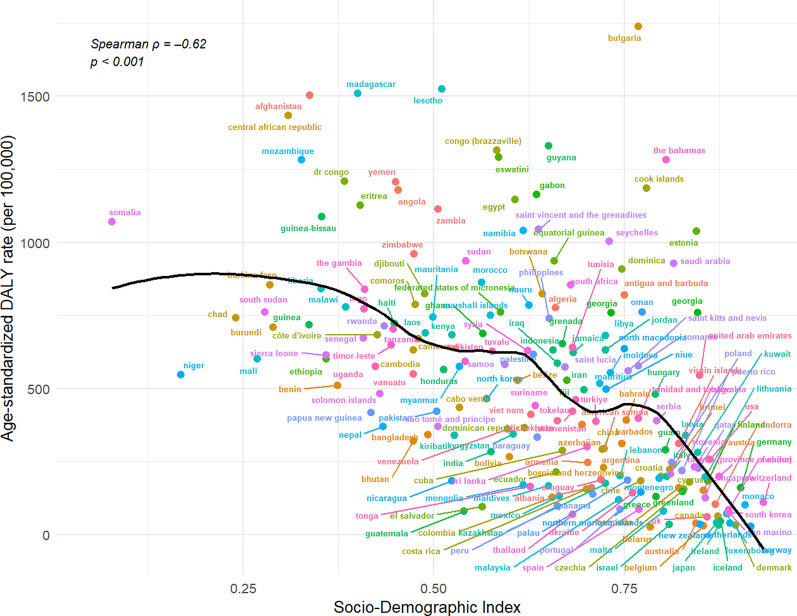
Relationship between Socio-demographic index (SDI) and hypertensive heart disease (HHD) burden in 2021. Scatterplot showing the association between age-standardized DALY rates for HHD and SDI across 204 countries in 2021. The LOESS curve illustrates a nonlinear inverse relationship (Spearman ρ = – 0.62; *p* < 0.001)

As shown in Fig. 2, the age-standardized DALY rate for HHD in 2021 displayed a clear inverse and nonlinear relationship with the socio-demographic index (SDI). Countries with SDI values below 0.50—such as the Central African Republic—reported DALY rates exceeding 1,000 per 100,000 population. South Sudan and several other low-SDI countries also exhibited rates well above the global average.

 The LOESS curve showed a sharp decline in DALY rates between SDI scores of 0.30 and 0.70, with the curve flattening at higher SDI levels. This trend was supported by a Spearman correlation coefficient of – 0.62 (*p* < 0.001). A few countries deviated from the overall pattern; for instance, the Cook Islands recorded a substantially higher DALY rate compared to other countries with similar SDI values. These observed discrepancies indicate that variation exists beyond the primary SDI gradient.

### Future projections of DALY burden (2022–2036)

**Fig. 3:**
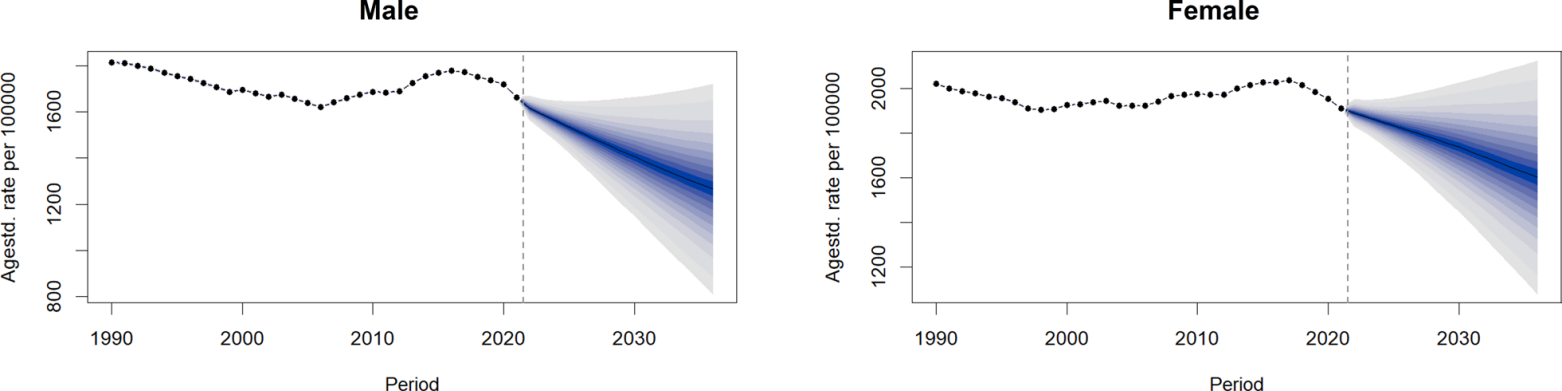
Global trends and Bayesian projections of age-standardized DALY rates for hypertensive heart disease (HHD) by sex, 2022–2036. Observed trends in age-standardized DALY rates (1990–2021) and Bayesian age–period–cohort (BAPC) model projections (2022–2036), with 95% uncertainty intervals, stratified by sex. Females are projected to maintain higher DALY rates and exhibit greater forecast uncertainty compared to males

Based on BAPC modeling, the global age-standardized DALY rate for HHD is projected to remain relatively stable through 2036, with a slight overall downward trend (Fig. 3). From 1990 to around 2010, the rate declined consistently, followed by a plateau in recent years. Sex-stratified projections indicate persistently higher age-standardized DALY rates in females, with median estimates ranging from approximately 1800 to 2000 per 100,000 population, compared to 1200 to 1600 in males. Additionally, the 95% credible intervals for females are wider, suggesting greater statistical uncertainty in future estimates for women.

### Decomposition and risk factor attribution analysis

**Fig. 4:**
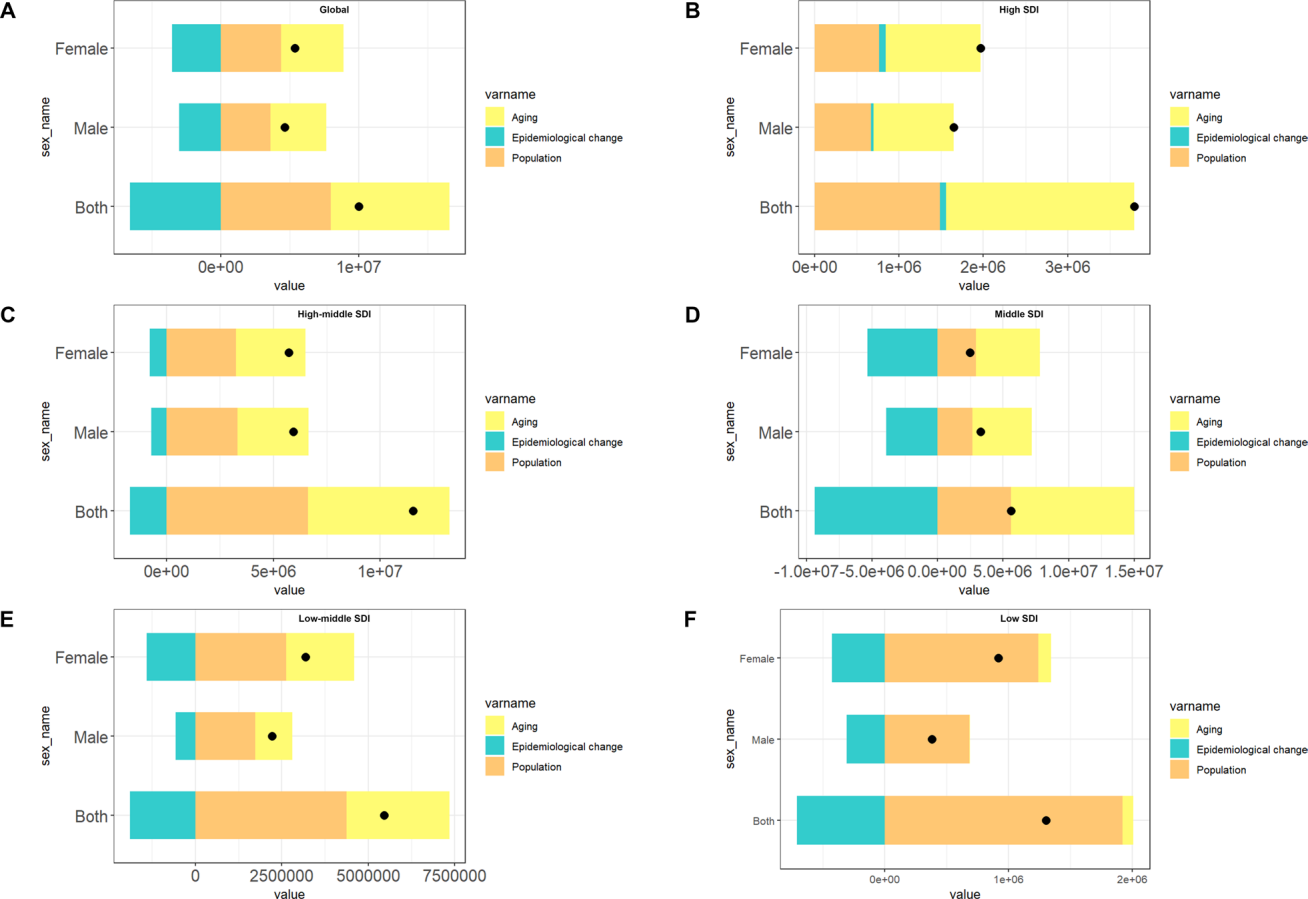
Decomposition of changes in HHD-related DALYs into demographic and epidemiological drivers by sex and SDI level. Decomposition of changes in HHD-related DALYs from 1990 to 2021 into contributions from population growth, population aging, and epidemiological change, stratified by SDI level and sex. Negative values represent reductions due to epidemiological improvements

The decomposition analysis partitioned the net change in HHD-related DALYs from 1990 to 2021 into three contributing components: population growth, population aging, and epidemiological change (Fig. 4). Population growth emerged as the predominant contributor to the overall increase in DALYs, particularly in low- and middle-SDI regions. In high-SDI settings, population aging assumed a comparatively greater role, especially among females. Epidemiological change, which reflects shifts in age-specific rates due to improvements in prevention or treatment, resulted in modest reductions in certain subgroups.

**Fig. 5:**
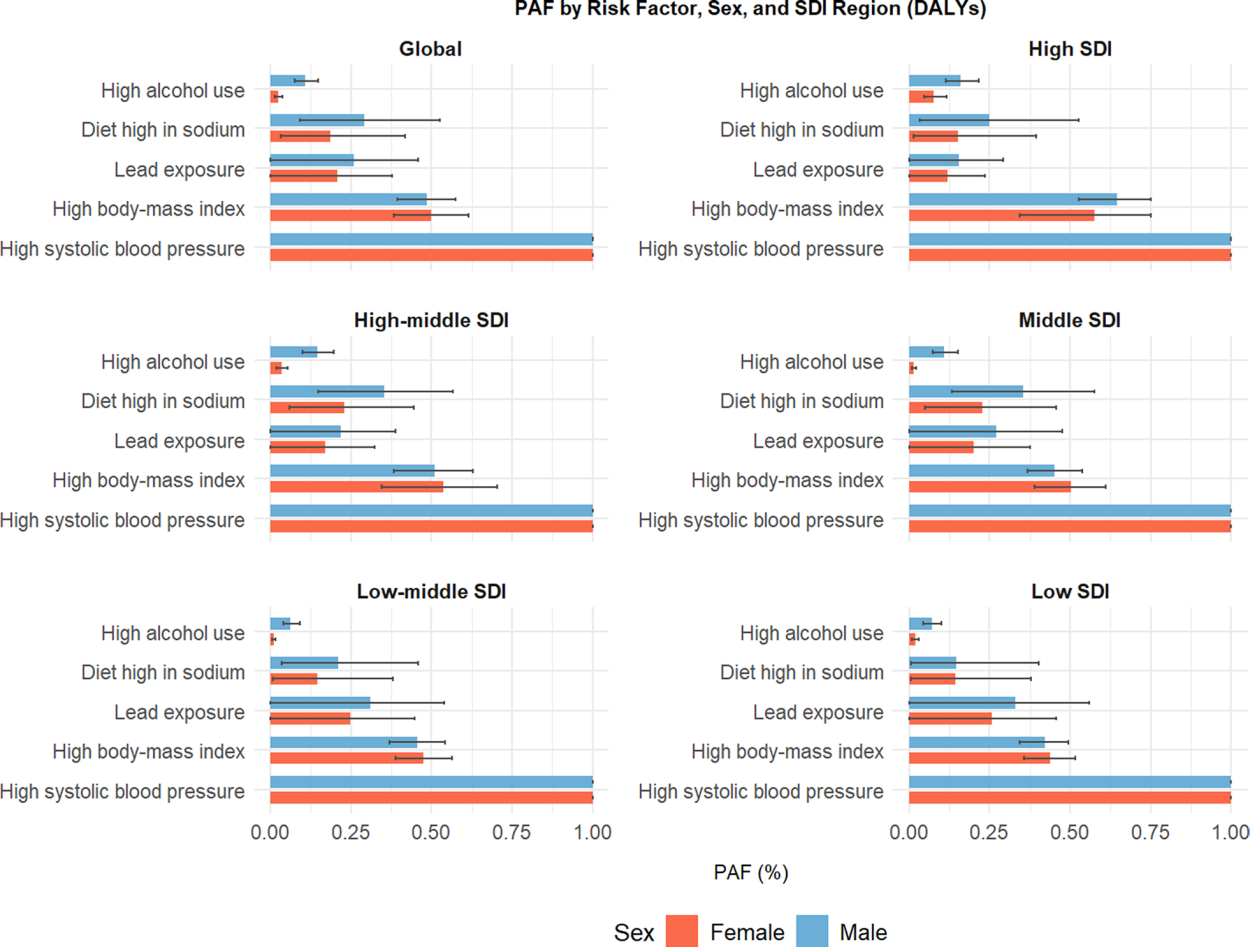
Top five modifiable risk factors for HHD-related DALYs by sex and SDI region in 2021. Population-attributable fractions (PAFs) of HHD-related DALYs in 2021 for five modifiable risk factors, stratified by SDI region and sex. High systolic blood pressure (SBP) was the dominant contributor across all strata. High body mass index (BMI) had a greater impact among females, while high sodium intake contributed more among males. Lead exposure surpassed high sodium intake in low-SDI males. High alcohol use had minimal impact, primarily in men

In the risk factor attribution analysis, five modifiable exposures were evaluated: high SBP, high BMI, high dietary sodium intake, high alcohol use, and lead exposure (Fig. 5). High SBP emerged as the predominant contributor across all SDI levels and both sexes. High BMI exhibited a more substantial contribution in females, while high sodium intake consistently ranked third, with higher population attributable fractions (PAFs) observed in males, particularly within middle- and high-SDI groups. Notably, lead exposure exceeded high sodium intake in low- and low-middle SDI males. High alcohol consumption had a minimal impact on females but was more pronounced in males across all SDI strata.

## Discussion

This study delineates global patterns, temporal trends, and socioeconomic disparities in age-standardized DALY rates attributable to HHD from 1990 to 2021. While the age-standardized DALY rate declined in many countries—especially those in the high-SDI group—our Joinpoint regression revealed that the rate of decline varied over time and across SDI levels. Despite these improvements, the global number of HHD-related DALYs increased by over 60% during the study period, driven largely by population growth and aging. Notably, countries with lower socioeconomic development continued to bear a disproportionately high burden, and pro-poor disparities appeared to widen over time. In low- and middle-SDI settings, absolute DALYs rose steadily, even where age-standardized DALY rates showed only modest improvement or remained stable. Additionally, the burden among women increased despite plateauing age-standardized DALY rates suggesting growing gender-based inequality. These findings point to uneven progress in hypertension prevention, diagnosis, and management globally, underscoring the urgent need for equity-focused and sex-responsive cardiovascular interventions.

Our study found that high SBP remained the leading modifiable risk factor for HHD-related DALYs across all SDI levels and sex groups, consistent with previous evidence linking elevated SBP to myocardial remodeling and progression to heart failure  [23–25]^.^ Stratified comparative risk assessment revealed distinct subgroup-specific patterns of attributable burden. Among females in high-SDI regions, high BMI contributed more substantially to HHD burden, consistent with studies reporting greater cardiometabolic susceptibility to central adiposity and a higher prevalence of diastolic dysfunction in women  [26, 27]. These differences may be partly mediated by sex-specific mechanisms involving metabolic inflammation and insulin resistance, as suggested by prior mechanistic research  [28]. In contrast, lead exposure and high alcohol use contributed more to the burden among males in low- and lower-middle SDI regions, which is consistent with global estimates indicating that men in socioeconomically disadvantaged populations face higher occupational and behavioral risk exposures [29, 30]. High sodium intake consistently ranked among the top contributors in all subgroups, with a higher attributable burden in males, likely reflecting persistent sex- and region-based disparities in dietary sodium consumption and its cardiovascular effects  [31, 32]. These findings underscore the influence of socio-demographic context on differential exposure to metabolic, behavioral, and environmental risk factors, contributing to the heterogeneity of HHD burden across populations.

Our findings are consistent with recent GBD-based analyses indicating a rising burden of HHD among females, particularly in low-SDI regions  [33]. This trend may reflect a combination of biological vulnerability and systemic inequities in healthcare access. In many resource-limited settings, women are more likely to experience delayed diagnosis, inadequate treatment access, and limited access to specialized cardiovascular services [34]. Hormonal changes associated with menopause—particularly estrogen decline—have been linked to increased arterial stiffness and impaired diastolic function, which may elevate cardiometabolic risk in aging women  [35]. From a demographic standpoint, our decomposition analysis revealed that population growth was the primary contributor to increasing HHD-related DALYs in low- and middle-SDI countries, whereas population aging played a more dominant role in high-SDI settings. These findings align with broader global demographic shifts reported in previous GBD assessments  [36, 37]. In middle-SDI regions, the modest decline in age-specific DALY rates may reflect gradual improvements in hypertension awareness, treatment coverage, and healthcare delivery. These subgroup-specific trends were quantitatively confirmed through decomposition analysis, which distinguished the relative contributions of population growth, aging, and age-specific burden across SDI strata.

The observed disparities in HHD burden across countries highlight persistent global health inequities. Despite notable advancements in cardiovascular disease prevention and management, low-SDI countries continue to shoulder a disproportionate burden—primarily due to weak healthcare infrastructure, limited access to antihypertensive treatment, and underinvestment in primary care services  [38]. Our findings reveal a curvilinear inverse association between SDI and age-standardized DALY rates, suggesting that socioeconomic progress alone does not ensure equitable cardiovascular outcomes. Substantial differences in HHD burden were evident even among countries with similar SDI levels, likely reflecting variation in national health financing, the availability of trained healthcare personnel, the strength of public health systems, and underlying sociocultural factors  [39]. These results emphasize that biomedical interventions, while essential, are insufficient to close the equity gap in HHD outcomes. Structural determinants of inequality—including resource-constrained health systems, gender-based barriers, and geographic disparities in service delivery—must be addressed through broader, multisectoral strategies. Moreover, our long-term projections support the implementation of gender-sensitive and context-specific interventions to improve hypertension detection, treatment adherence, and long-term management, particularly in regions with persistent resource limitations  [40].

Our findings have significant public health implications. The rising absolute burden of HHD, despite declining age-standardized DALY rate, underscores the need to strengthen population-level prevention efforts, such as improving hypertension screening and promoting lifestyle interventions to reduce modifiable risk factors (e.g., high BMI, high sodium intake, high alcohol use). Additionally, the pronounced burden among women and in low-SDI regions suggests that policies should prioritize equity-focused resource allocation and capacity building in primary care and hypertension management. Targeted interventions, including community-based education, affordable medication access, and culturally tailored health promotion programs, are essential to address the disproportionate risk in vulnerable populations. These efforts should be integrated into broader national and regional non communicable disease strategies to effectively reduce HHD burden and improve cardiovascular health equity worldwide.

This study provides a timely and comprehensive evaluation of the global burden of HHD, based on standardized estimates from the Global Burden of Disease 2021 study. By combining EAPC analysis, BAPC modeling, and decomposition techniques, we examined long-term trends and quantified the respective contributions of population growth, aging, and changes in age-specific DALY rates. Stratification by SDI and sex enhanced the granularity of our findings and their relevance for policy and equity-focused interventions. The methodological rigor and global comparability of GBD data further strengthen the reliability and generalizability of our results.

However, several limitations should be considered. First, the quality of input data varies across countries, particularly in low-SDI settings where health surveillance systems are often less developed. Second, the BAPC forecasting model is based on historical trends and may not capture future changes in health policies or intervention uptake. Third, the use of aggregated, population-level data limits the ability to draw individual-level inferences. Additionally, other important dietary risk factors such as low intake of fruits and vegetables and consumption of processed meat or whole grains were not included in this analysis. These challenges are common in global burden analyses and underscore the importance of strengthening health information systems and integrating longitudinal, individual-level data in future research. Nevertheless, these limitations do not detract from the overall validity of our findings or their implications for global health policy.

Future research should address several critical gaps. First, improving data quality and strengthening surveillance systems in low-SDI settings are essential to produce more accurate burden estimates and enable timely monitoring. Second, further studies are needed to elucidate sex-specific causal mechanisms underlying hypertensive heart disease, including biological, behavioral, and social determinants. Third, rigorous evaluations of both preventive and therapeutic interventions are required to assess their effectiveness and cost-effectiveness, particularly in resource-limited contexts. Finally, incorporating more granular subregional or subnational data will help capture local variations and guide targeted, equity-oriented public health strategies.

## Conclusion

This study provides a global evaluation of the burden of HHD across diverse socio-demographic contexts. Although age-standardized DALY rates have declined, the absolute burden has continued to grow—driven primarily by population aging and growth. Persistent disparities across SDI levels and between sexes highlight the need for equity-focused strategies, particularly in resource-limited settings. Strengthening hypertension screening, sustaining long-term risk control, and developing region-specific predictive models and mechanistic insights will be essential for reducing the global impact of HHD.

## Supplementary Information

Supplementary Material 1.

## Data Availability

https://ghdx.healthdata.org/gbd-2021

## Abbreviations

APC: Annual percentage change
AAPC: Average annual percentage change
BAPC: Bayesian age–period–cohort model
BMI: Body mass index
DALYs: Disability-adjusted life years
EAPC: Estimated annual percentage change
GBD: Global burden of disease
HHD: Hypertensive heart disease
PAF: Population-attributable fraction
SBP: Systolic blood pressure
SDI: Socio-demographic index
