# Supplementary material for: Sociodemographic and sex differences in the global burden of hypertensive heart disease, 1990–2021: a population-based analysis
Source: Trop Med Health. 2025 Aug 14;53:108. doi: 10.1186/s41182-025-00791-9 (PMC12351887; doi:10.1186/s41182-025-00791-9)
Supplement: Supplementary file 1 — Supplementary Material 1. [file 41182_2025_791_MOESM1_ESM.docx]

Table S1 Member countries and territories within each GBD super region (GBD 2021 classification)

| GBD super region | Countries and territories |
| --- | --- |
| Central Europe, Eastern Europe, and Central Asia | Albania, Armenia, Azerbaijan, Belarus, Bosnia and Herzegovina, Bulgaria, Croatia, Czech Republic, Estonia, Georgia, Hungary, Kazakhstan, Kyrgyzstan, Latvia, Lithuania, Moldova, Montenegro, North Macedonia, Poland, Romania, Russia, Serbia, Slovakia, Slovenia, Tajikistan, Turkmenistan, Ukraine, Uzbekistan. |
| High-income | Andorra, Australia, Austria, Belgium, Canada, Cyprus, Denmark, Finland, France, Germany, Greece, Iceland, Ireland, Israel, Italy, Japan, Luxembourg, Malta, Monaco, Netherlands, New Zealand, Norway, Portugal, Singapore, South Korea, Spain, Sweden, Switzerland, United Kingdom, United States. |
| Latin America and Caribbean | Argentina, Bahamas, Barbados, Belize, Bolivia, Brazil, Chile, Colombia, Costa Rica, Cuba, Dominican Republic, Ecuador, El Salvador, Guatemala, Guyana, Haiti, Honduras, Jamaica, Mexico, Nicaragua, Panama, Paraguay, Peru, Puerto Rico, Suriname, Trinidad and Tobago, Uruguay, Venezuela. |
| North Africa and Middle East | Afghanistan, Algeria, Bahrain, Egypt, Iran, Iraq, Jordan, Kuwait, Lebanon, Libya, Morocco, Oman, Palestine, Qatar, Saudi Arabia, Sudan, Syria, Tunisia, Turkey, United Arab Emirates, Yemen. |
| South Asia | Bangladesh, Bhutan, India, Nepal, Pakistan. |
| Southeast Asia, East Asia, and Oceania | Brunei, Cambodia, China, Fiji, Indonesia, Laos, Malaysia, Maldives, Mongolia, Myanmar, North Korea, Papua New Guinea, Philippines, Samoa, Solomon Islands, South Korea, Sri Lanka, Taiwan, Thailand, Timor-Leste, Tonga, Vanuatu, Vietnam. |
| Sub-Saharan Africa | Angola, Benin, Botswana, Burkina Faso, Burundi, Cameroon, Cape Verde, Central African Republic, Chad, Comoros, Republic of the Congo, Democratic Republic of the Congo, Equatorial Guinea, Eritrea, Eswatini, Ethiopia, Gabon, Gambia, Ghana, Guinea, Guinea-Bissau, Ivory Coast, Kenya, Lesotho, Liberia, Madagascar, Malawi, Mali, Mauritania, Mauritius, Mozambique, Namibia, Niger, Nigeria, Rwanda, São Tomé and Príncipe, Senegal, Seychelles, Sierra Leone, Somalia, South Africa, South Sudan, Togo, Uganda, United Republic of Tanzania, Zambia, Zimbabwe. |
